# Editorial

**DOI:** 10.1177/09544119211028504

**Published:** 2021-07-02

As you will be aware I only write Editorials infrequently. For example, around the turn of the year I like to thank all our colleagues, Authors, Reviewers, members of the Editorial Board and the staff at SAGE for all their hard work during the year. I especially like to thank our readers for their continuing support. However the 30th June 2021 is a very significant day for me personally as I shall cease to be your Editor in Chief at the end of that day after twenty eight and a half years in the role.

It has been an incredible privilege for me to have spent so long with you and I want to thank you all most sincerely for allowing me to share this time with you.

I became Editor-in-Chief of *Journal of Engineering in Medicine* on the 1st January 1993 and since that time we have increased our issues from 4 to 12/year and have expanded our international authorship magnificently. I took over the Editor’s role from my friend and Mentor, Professor Duncan Dowson who, to my delight, remained as a Board member until his passing in January 2020. Duncan and I exchanged telephone calls regularly and he always asked about the Journal which remained so very dear to him.

However it is now time for the Journal to move onward and upward and I am delighted to welcome my successor, Professor Liz Tanner who has been a very active Associate Editor with Part H for many years and I trust that you will give her the loyal support that you kindly gave to me. Publishing is changing dramatically and as I hand over to Professor Tanner I am delighted to wish her and all of you, every success for the future.


**Anthony Unsworth** Durham University, Durham, UK


