# Supplementary material for: Editorial
Source: Proc Inst Mech Eng H. 2021 Jul 2;235(9):975. doi: 10.1177/09544119211028504 (PMC8640262; doi:10.1177/09544119211028504)
Supplement: Related texts

# Inaugural editorial

**Published:** 2021-07-02

**Authors:** Elizabeth Tanner

It is a privilege to take on the Editorship of *Journal of Engineering in Medicine* from Tony Unsworth. To serve for nearly 30 years as Editor of a journal was unusual even in the first part of the 20th Century, to do so in the late 20th and early 21st Century must be nearly unique. My first, and very pleasant duty, is to announce that Tony is not really leaving the Journal, merely becoming the Emeritus Editor. The *Journal of Engineering in Medicine* has been special to me since my first paper was published with my status given as ‘Undergraduate’ when Bernard Lucas the founding editor and Duncan Dowson were the Editors. I am really honoured and delighted to take on the mantle of Bernard Lucas, Tony and Duncan. After 50 years of the Journal to be the fourth Editor is an honour.

We have to thank Tony for his efforts bringing the journal from the days of paper and physical photograph submissions to the Institution of Mechanical Engineers to today’s electronic submission and review with our publisher SAGE. I would like to thank the Editorial Board and Associate Editors for the support they have given the Journal over the years since I joined the Editorial Board. I know I will require their expertise and support as I take on this job.


**Elizabeth Tanner** Queen Mary University of London, London, UK
